# Motor Performance in Children Diagnosed with Cancer: A Longitudinal Observational Study

**DOI:** 10.3390/children7080098

**Published:** 2020-08-15

**Authors:** Lotta Hamari, Päivi M. Lähteenmäki, Heidi Pukkila, Mikko Arola, Anna Axelin, Sanna Salanterä, Liisa S. Järvelä

**Affiliations:** 1 Department of Nursing Science, University of Turku, 20014 Turku, Finland; anmaax@utu.fi (A.A.); sansala@utu.fi (S.S.); 2 Turku University Hospital, P.O. Box 52, 20521 Turku, Finland; 3 Department of Pediatrics and Adolescent Medicine, Turku University Hospital, P.O. Box 52, 20521 Turku, Finland; paivi.maria.lahteenmaki@tyks.fi (P.M.L.); liisa.jarvela@utu.fi (L.S.J.); 4 Department of Clinical Medicine, University of Turku, 20014 Turku, Finland; 5 Faculty of Engineering and Natural Sciences, Tampere University, Korkeakoulunkatu 6, 33720 Tampere, Finland; pukkilah@gmail.com; 6 Department of Pediatrics, Tampere University Hospital, Elämänaukio, Kuntokatu 2, 33520 Tampere, Finland; mikko.arola@pshp.fi

**Keywords:** childhood cancer, motor performance, physical activity, motor activity, longitudinal study

## Abstract

Children with cancer are dealing with different side and long-term effects caused by cancer and its treatments, like vinca-alkaloids, which may have negative effects on motor performance. However, the affected areas of motor performance (aiming and catching, balance, manual dexterity) and the differences in these areas between boys and girls and diagnoses are not frequently reported in a longitudinal design. Therefore, the aim of this study was to investigate how motor performance changes over the course of cancer treatment. The study was conducted with 3-to 16-year-old children with cancer (N = 36) in 2013–2017. The five assessment points were 0, 2, 6, 12 and 30 months from diagnosis. Movement-ABC2 was used to assess motor performance. We found that aiming and catching skills decreased significantly during the follow-up (*p* < 0.05). Balance was affected at the 2-month measurement point (*p* < 0.05) and more in children with acute lymphoblastic leukemia than in children with other cancer diagnoses (*p* < 0.05). Girls performed better than boys in manual dexterity at 6, 12 and 30 months (*p* < 0.05, *p* < 0.05, *p* < 0.05, respectively). Individual monitoring of motor performance with standardized tests and physical activity/exercise programs during and after treatment are needed.

## 1. Introduction

Children diagnosed with cancer have to deal with different side and long-term effects [1,2] caused by cancer and its intensive treatments. The negative effects during and after treatment may include fatigue, obesity, reduced cardiopulmonary capacity, decreased musculoskeletal health and motor performance, and decreased health-related quality of life [2,3,4,5,6,7]. All of these may be influenced by physical activity during and after treatment [8,9,10,11,12,13].

The number of studies published regarding physical activity during and after treatment of childhood cancer has greatly increased since the 1990s when the first article [14] was published [10]. This means that the interest as well as also importance and evidence base of physical activity in children with cancer have grown enormously. Exercise and motor interventions are reported to improve physical activity levels, functional mobility, range of motion, muscle strength, coordination, bone mineral density, aerobic capacity, quality of life, self-efficacy, as well as neurocognitive functioning, and to reduce fatigue with no adverse effects reported [10,15,16].

However, not many hospitals globally are offering in-hospital physical activity programs to pediatric cancer population [17]. In addition, the program characteristics vary greatly [17]. To inform hospitals, community organizations and those professionals that are offering physical activity programs to children diagnosed with cancer, more information is needed about the timing and factors in motor performance. When developing and offering those programs, it is needed to know what kind of exercises to focus on at each stage of the treatment. Furthermore, one study that reviewed empirical studies examining motor skills in children during and after treatment for acute lymphoblastic leukemia [6], suggested that longitudinal designs to specify the timing and onset of motor difficulties and associated factors should be carried out. Therefore, the aim of the present study was to describe how motor performance changes longitudinally over the course of the treatment regimen in children diagnosed with cancer. The conclusions drawn by this study will help in developing effective physical activity programs that support motor performance with the right focus on its domains within different subgroups.

In this study, we report changes in motor performance during cancer treatment in children and compare the performance in different motor performance domains between girls and boys, and between children with acute lymphoblastic leukemia (ALL) and with other diagnoses.

## 2. Subjects and Methods

This study was conducted as a longitudinal observational study that includes secondary analyses of a randomized controlled trial [18,19]. The detailed methods are reported in Kauhanen et al., 2014 [19], and those relating to the present study are repeated here. The ethical approval was received from the Joint Commission on Ethics of the Hospital District of South-West Finland (15 May 2012 § 153). The research approvals were obtained from both participating institutions (24 September 2012 K66/12 No. 13059 and 21 March 2013 65 § R13030). All study participants have given an informed consent to participate in this study.

### 2.1. The Research Questions

(1) How does motor performance change within its different domains from the beginning of the treatment to 2, 6, 12 and 30 months from the diagnoses?
(2) What are the differences in motor performance domains between boys and girls and within different cancer diagnoses?

### 2.2. Participants

**Figure 1:**
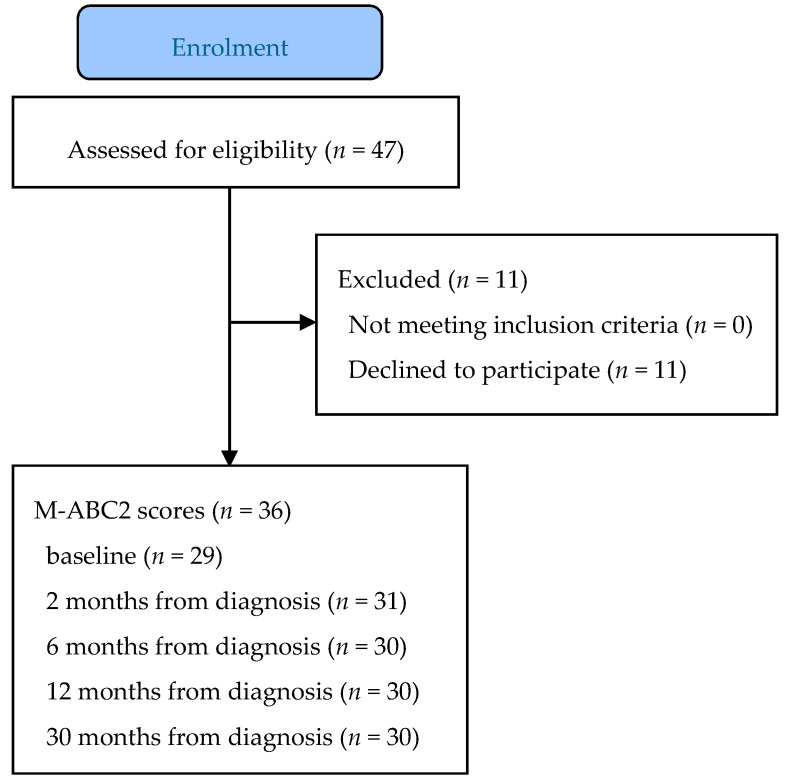
Flow chart of participants.

The study population consisted of 3- to 16-year-old children diagnosed with cancer outside the central nervous system (including acute lymphocytic leukemia, Hodgkin and non-Hodgkin lymphomas, neuroblastoma, Wilms tumor, rhabdomyosarcoma, retinoblastoma, and Ewing sarcoma). The eligibility criteria were as follows: (1) aged 3 to 16 years at the time of diagnosis; (2) cancer treatment with a protocol including vincristine, and (3) treated in either of the two designated hospitals (Turku University Hospital or Tampere University Hospital, Finland). After the screening phase, the researcher met the potential participants and gave the detailed research information to all patients meeting the eligibility criteria. Participant flow is reported in Figure 1. The early-phase intervention in the randomized controlled trial (RCT) study showed not to be effective, and therefore, the groups were combined for the follow-up and secondary analyses [18].

### 2.3. Outcome Measurement: Motor Performance

The Movement Assessment Battery for Children-2 (M-ABC2) [20] was used to measure motor performance. The M-ABC2 is as a valid instrument to measure motor performance in this population [7,21]. The measurement points were 0, 2, 6, 12 and 30 months from the diagnosis. The tests were conducted by a physiotherapist at the hospital during the routine appointments or inpatient stays.

The M-ABC2 contains tasks for manual dexterity (MD), aiming and catching (AC), and balance (BAL), and also, three different test sets for age ranges: 3–6 years; 7–10 years and 11–16 years. For 3- to 6-year-old children the tasks contain posting coins, threading beads, drawing trail, catching and throwing a beanbag, one-leg balance, walking heals raised, and jumping on mats. For 7- to 10-year-old children the tasks contain placing pegs, threading lace, drawing trail, catching and throwing beanbag, one-board-balance, walking heal-to-toe forwards, and hopping on one leg. For 11- to 16-year-old children the tasks contain turning pegs, triangle with nuts and bolts, drawing trail, catching and throwing with one hand, throwing ball at a wall target, two-board balance, walking toe-to-heel backwards, and zig-zag hopping with one leg. In this study, the test set was chosen each time based on the child’s age at the test day.

The motor performance scores are presented in percentiles and a higher number means better performance. The child is at risk of having movement difficulties if the percentile score is ≤15. The percentile below 5 indicates significant motor difficulty [20].

### 2.4. Data Analysis

The motor performance scores from the intervention and control groups were combined in the analyses of this study, since we did not find any differences between the groups on pre-test, post-test or change in pre- and post-test measurements in the previous RCT study [18].

The differences in M-ABC2 percentiles between time points, genders and diagnoses were tested using the Wilcoxon signed rank test and Wilcoxon rank sum test due to skewed distribution of data. The level of statistical significance was set at *p* < 0.05. Only those participants who had the baseline data were included in the analyses. In the descriptive statistics (Table 1) data from all participants are reported. Statistical analyses and figures were performed using R-language (R 2.13.2).

## 3. Results

### 3.1. Demographics

A total of 36 children diagnosed with cancer agreed to participate (Table 1). Detailed demographics of the sample are reported in Hamari et al. 2019 [18].

### 3.2. Change in Motor Performance Domains during Treatment

**Figure 2:**
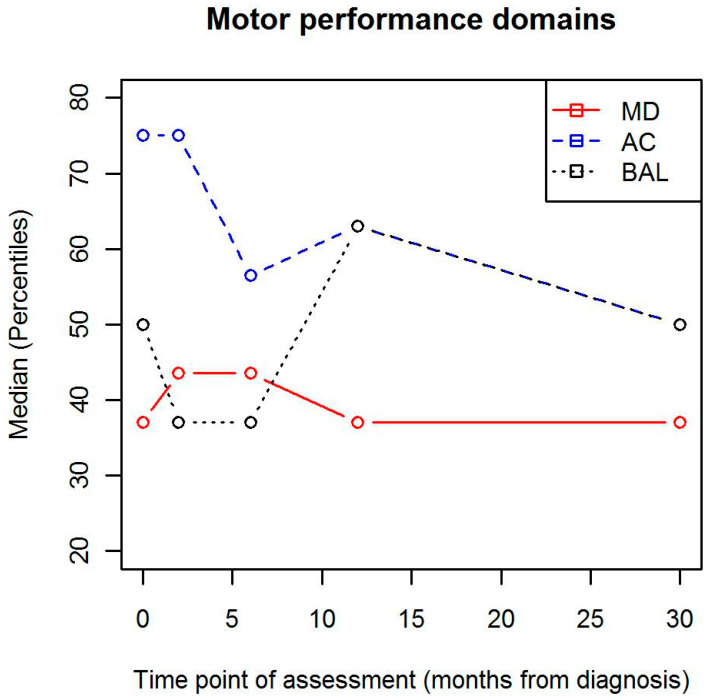
Changes in motor performance percentiles during treatment. MD = manual dexterity; AC = aiming and catching; BAL = balance.

The change in motor performance domains (aiming and catching, balance, manual dexterity) are illustrated in Figure 2, and the descriptive values are presented in Table 2. Of the three domains, aiming and catching (AC) scores decreased significantly between the baseline (the time of diagnosis) and the last (30 month) measurement point (median, interquartile range (IQR), 75 (45) vs. 50 (52.25), respectively, *p* = 0.047). Balance (BAL) was affected the most at the 2-month (median (IQR), 37 (54), *p* = 0.008) measurement point, when compared to the 12-month (median (IQR), 50 (66)) measurement point. The differences between the scores at different time point were not significant regarding manual dexterity (MD) in any comparisons.

### 3.3. Difference in Motor Performance between Different Diagnoses

**Figure 3:**
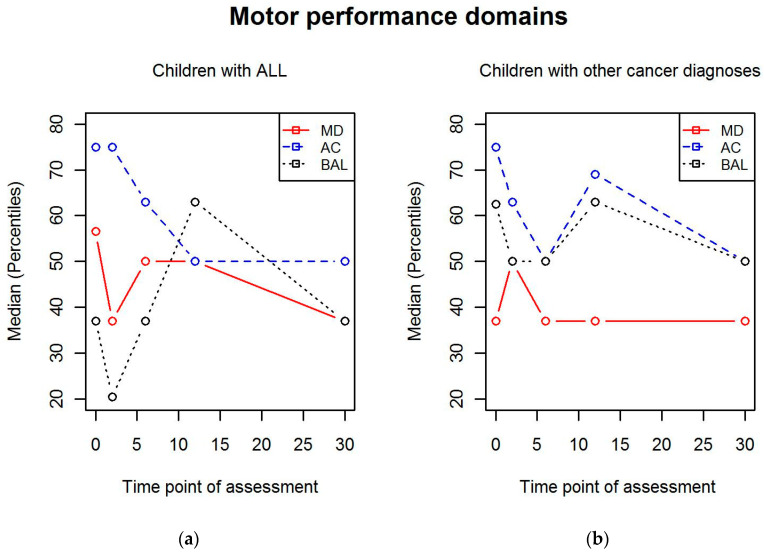
The changes in the different domains of motor performance during treatment in (**a**) children with acute lymphoblastic leukemia (ALL) and (**b**) children with other cancer diagnoses. MD = manual dexterity; AC = aiming and catching; BAL = balance.

When children with ALL and children with other cancer diagnoses were analyzed separately, the difference in balance scores between the 2- and 12-month measurement points (*p* = 0.016) and 6- and 12-month measurement points (*p* = 0.008) was only seen in the group with children with ALL. In addition, scores were significantly different between 2-month and 30-month measurement points (*p* = 0.025) in children with ALL. The changes in the different domains of motor performance in children with ALL and children with other cancer diagnoses are illustrated in Figure 3.

### 3.4. Differences between Gender in Motor Performance

**Figure 4:**
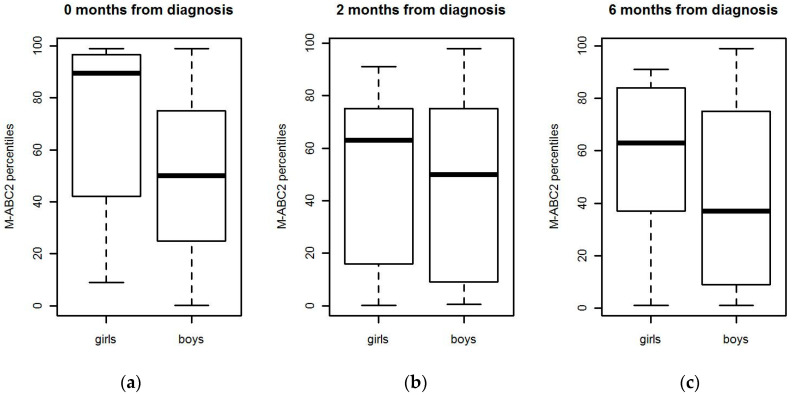

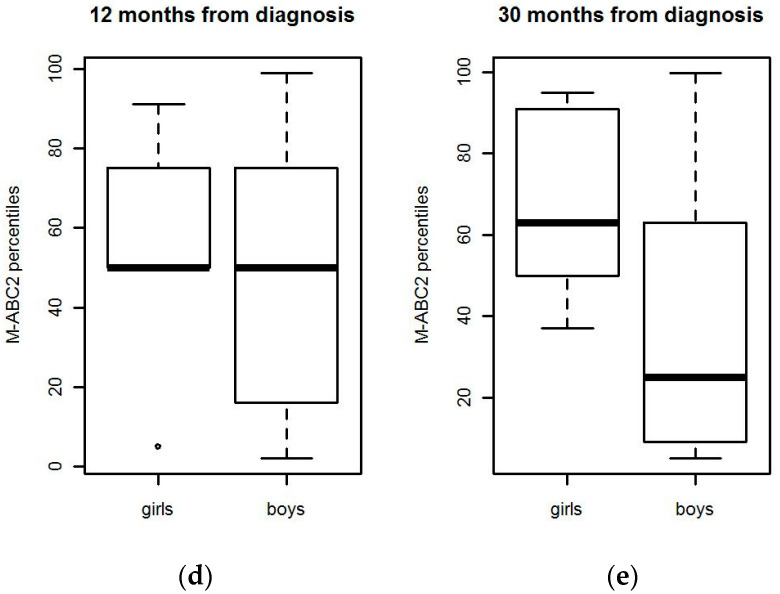
Motor performance scores for boys and girls in (**a**) 0, (**b**) 2, (**c**) 6, (**d**) 12, and (**e**) 30 months.

The difference between boys and girls was not statistically significant at any time point (Figure 4).

**Figure 5:**
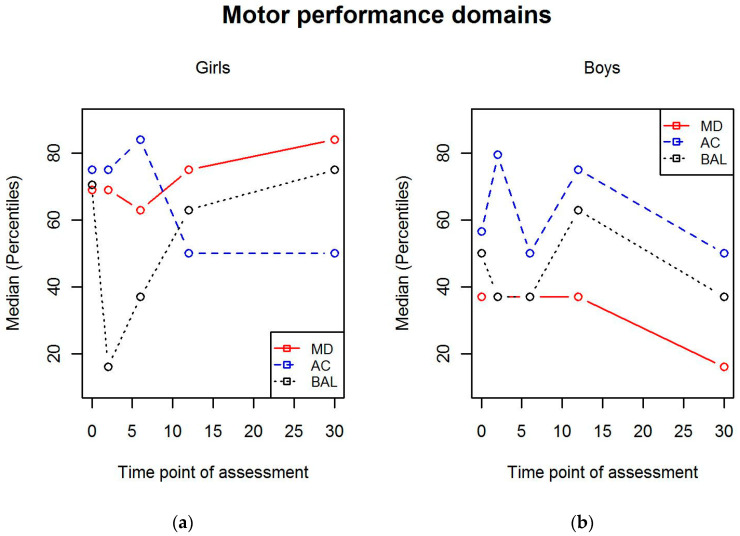
Scores for motor performance domains in (**a**) girls and (**b**) boys. MD = manual dexterity; AC = aiming and catching; BAL = balance.

When the different domains of motor performance were compared between boys and girls, we found that manual dexterity skills were better in girls at 6-, 12-, and 30-month measurement points (*p* = 0.033, *p* = 0.036, *p* = 0.002, respectively). There were no statistical differences in aiming and catching or balance between genders. The motor performance domains in girls and boys separately are illustrated in Figure 5.

## 4. Discussion

The main findings of this study were that aiming and catching (i.e., ball skills) decreased significantly during the 30 month follow-up period after cancer diagnosis in children, and balance was affected the most at 2 months after cancer diagnosis. Balance was more affected in children with ALL than in children with other cancer diagnoses. We did not find differences between time points in manual dexterity scores; however, the performance in manual dexterity skills remained low during the whole study period (median still remaining at the normal range of motor skills). Girls performed better than boys in manual dexterity at the 6-, 12- and 30-month measurement points. It is noteworthy that the motor performance scores in the group of children with other cancer diagnoses did not improve from the 12 to 30 month time points, even though the treatment had already ended for most of them at 6 or 12 months, as the treatment for other childhood malignancies is much shorter than for ALL.

The limitations of this study are the small sample size and the heterogeneity of the sample regarding different ages. We also recognize the missing sample size calculations as a limitation, and therefore, our patient number might have been too low for detecting all the possible differences between the subgroups. The number of girls and boys in our sample was also unbalanced, as we only had 10 girls vs. 26 boys. This may affect the generalizability of our results. The strength of this study instead is the long follow-up period, which enabled us to illustrate the changes in motor performance during different phases of cancer treatment in children.

With regard to deficits in motor performance, our results are in accordance with previous findings during cancer treatment [6,9,22]. The significant decrease in aiming and catching (ball skills) during the follow-up may be related to the general clumsiness caused by the use of vinca-alkaloids such as vincristine. However, these skills can also be easily practiced. Balance was affected in children with ALL at the beginning of treatment, and this may illustrate the general condition of the patients, as leukemia is a disease of the whole body, whereas other malignancies are mostly quite local; and within this group, neither of them had any central nervous system (CNS) manifestations. The balance of ALL patients improved remarkably during the first year, after the lowest point at two and six months after diagnosis. Most likely this reflects the course of ALL treatment, the most intensive treatment phases being during the first 6 months after diagnosis. The drop towards the end of treatment is difficult to explain but may, on the other hand, be a reflection of the whole burden of long-lasting cancer therapy as well as the effects of vincristine on the peripheral nerves causing, for example, loose ankles/foot drop, loss of ankle range of motion and muscle strength [23]. The long-term effect of vinca-alkaloids could be the explanation for the decreasing balance scores in the non-ALL group after the 12-month time point. One can also assume that the impairing performance in balance tests can, in the longer follow-up, be due to having missed a lot of normal age-associated motor learning possibilities due to both the hospitalizations during cancer treatment as well as related isolation practices leading to absence from school and organized sports. In addition, a well-known negative side-effect—cancer-related fatigue—may play a role in participation in motor activities during and after treatment [24,25,26]. Even though cancer-related fatigue may reduce the motivation to participate, it is important to encourage children to do so, since we know from a recent systematic review and meta-analysis that physical activity is effective in reducing fatigue in this population [15].

Regarding manual dexterity, De Luca et al. found that manual dexterity (measured with M-ABC2) was below the values of the normative population at 13 to 24 months-off-treatment in children with ALL [22]. They did not find gender differences on total test scores in M-ABC2. However, it must be noted that they included patients aged two-and-a-half to five years at the time of diagnosis and only children with ALL, so our samples are not fully comparable. In the healthy children’s population, findings suggest that motor performance differences between girls and boys vary depending on the type of task [27]. This was also evident in our study, as we found that girls performed better in manual dexterity. If we discuss this finding through traditional stereotypes, girls might be spending more time in fine motor activities, such as drawing, than boys [27]. This is, however, just speculation. The differences between genders may also be due to biological maturation differences in girls and boys at different ages.

Even though specialized physical therapy is many times needed and justified, other health care professionals, and especially parents, have a crucial role in maintaining a child’s physical functioning during treatment. Even in the patient rooms, it is possible to throw and catch soft inflatable balls with the child, when safety is guaranteed in surroundings. Wards could also have a special exercise place for practicing motor skills, including different equipment, like a little basketball hoop, painted hop-scotch on the floor, soft balancing pads, and painted targets on a wall for throwing and catching balls or beanbags. Balance can be practiced with balance boards, but also by doing different tasks by standing on one leg, reaching on objects or doing calf raises without support. Reaching on objects can be performed from standing or sitting position. In addition, many active video games challenge balance, and those can be played also in patient rooms, from standing or sitting position. Manual dexterity can be practiced for example by crafting, drawing or threading beads or lace. The M-ABC 2 tool kit (Pearson Education Ltd., Essex, London, UK, 2020), does have good examples of what kind of tasks could be offered to improve movement skills even during in-patient stays. Practicing at the hospital should be supervised by an adult in order to ensure safety. Specialized and suitable forms of practicing may be asked from a physical therapists or other exercise specialists that have been trained for the special needs and characteristics of the childhood cancer population. At home, families are recommended to be physically active in diverse ways and offer children versatile possibilities to be active within the limits given by the treating medical team. Family and siblings have an important role in children’s physical activity [28,29,30,31] and the time spent outdoors is positively associated with the amount of physical activity in young children [29,32]. The role of family in moving and playing with the child during treatment is emphasized since children cannot see their peers or take part in social activities during treatment because of the infection risks. It is worth remembering, that physical activity is extremely important in the prevention of many long-term side effects of cancer treatment, as for example, in reducing the risk of metabolic syndrome, cardiovascular diseases, and obesity in later in life.

Our findings support the fact that individual monitoring of motor performance with standardized tests conducted by rehabilitation professionals, and physical activity/exercise programs during and after cancer treatment in children are needed [6,9,10,22,23]. At least it would be important to have baseline values of their performance at the beginning of the treatment, to be able to evaluate the later effects and both the need for and effect of rehabilitation processes during and after the cancer treatment.

Our findings highlight the importance of physical activity in children with cancer from the very beginning of the treatment, to avoid a remarkable drop in their motor skills. Even though this study is focusing on a physical matter, i.e., motor performance, we want to highlight the importance of physical activity and its benefits also to the mental wellbeing of children. Reduced motor competence is one factor associated with lower preference for active play [33], and physical inactivity is associated with psychosocial wellbeing, cognitive functioning and educational achievement [34,35,36,37]. Keeping in mind these chains, it is easy to justify the need to focus on improving motor performance and motor activities during cancer treatment in children.

In the future, it would be beneficial to gather longitudinal data from a larger sample to confirm the results and to be able to conduct more subgroup analyses, for example to analyze children with different cancer diagnoses as their own subgroups, too.

## Figures and Tables

**Table 1:** The demographics of the study participants.

	N (%)	Age at Diagnosis (Median, Min–Max)
Study cohort	36 (100)	6.0 (3–16)
Girls	10 (28)	6.0 (4–16)
Boys	26 (72)	6.5 (3–15)
All	17 (47)	6.0 (3–16)
Standard risk	8	
Intermediate risk	6	
High risk	3	
Other	19 (53)	10 (3–15)
Wilms’ tumor	2	
Burkitt lymphoma	3	
Non-Hodgkin lymphoma	5	
Hodgkin lymphoma	3	
Other neoplasm	6	

**Table 2:** Descriptive values of movement ABC-2 percentiles during treatment (ALL = acute lymphoblastic leukemia).

	0 Months Median (IQR)	2 Months Median (IQR)	6 Months Median (IQR)	12 Months Median (IQR)	30 Months Median (IQR)
**Total**					
Study cohort	63 (64.5) *n* = 29	63 (66) *n* = 31	56.5 (68.25) *n* = 30	50 (59) *n* = 30	37 (50) *n* = 30
Boys	50 (54.5) *n* = 21	50 (69.25) *n* = 22	37 (66) *n* = 21	50 (59) *n* = 21	25 (54) *n* = 21
Girls	89.5 (71.75) *n* = 8	63 (67) *n* = 9	63 (56.5) *n* = 9	50 (29.5) *n* = 9	63 (49.5) *n* = 9
ALL	50 (87.5) *n* = 13	37 (63) *n* = 16	63 (75) *n* = 15	50 (59) *n* = 15	37 (50.5) *n* = 17
Other ^1^	63 (47) *n* = 16	63 (68) *n* = 15	50 (66) *n* = 15	63 (59) *n* = 15	63 (59) *n* = 13
**Aiming and catching**					
Study cohort	75 (45)	75 (66)	56.5 (70)	63 (47)	50 (52.25)
Boys	56.5 (37.25) *n* = 22	79.5 (71) *n* = 22	50 (70) *n* = 21	75 (48.75) *n* = 22	50 (44) *n* = 21
Girls	75 (42.5) *n* = 9	75 (58.5) *n* = 9	84 (51.5) *n* = 9	50 (53) *n* = 9	50 (59) *n* = 9
ALL	75 (52) *n* = 14	75 (57.75) *n* = 16	63 (58) *n* = 15	50 (47) *n* = 15	50 (50) *n* = 17
Other ^1^	75 (46) *n* = 17	63 (82) *n* = 15	50 (75) *n* = 15	69 (34) *n* = 16	50 (53) *n* = 13
**Balance**					
Study cohort	50 (66)	37 (54)	37 (55)	63 (57)	50 (66)
Boys	50 (66) *n* = 21	37 (54) *n* = 22	37 (54) *n* = 21	63 (62.5) *n* = 21	37 (56.5) *n* = 21
Girls	70.5 (74.75) *n* = 8	16 (72.45) *n* = 9	37 (51.5) *n* = 9	63 (54) *n* = 9	75 (54) *n* = 9
ALL	37 (72) *n* = 13	20.5 (31) *n* = 16	37 (32) *n* = 15	63 (66) *n* = 15	37 (75) *n* = 17
Other ^1^	62.5 (50.75) *n* = 16	50 (50) *n* = 15	50 (82) *n* = 15	63 (54) *n* = 15	50 (54) *n* = 13
**Manual dexterity**					
Study cohort	37 (52.25)	43.5 (69)	43.5 (54.5)	37 (66)	37 (68.25)
Boys	37 (50) *n* = 24	37 (70) *n* = 22	37 (37.5) *n* = 21	37 (42) *n* = 22	16 (32) *n* = 21
Girls	69 (52.25) *n* = 10	69 (46) *n* = 10	63 (41) *n* = 9	75 (34) *n* = 9	84 (28.5) *n* = 9
ALL	56.5 (65.75) *n* = 16	37 (74.5) *n* = 17	50 (58) *n* = 15	50 (59) *n* = 15	37 (74.5) *n* = 17
Other ^1^	37 (52.25) *n* = 18	50 (66) *n* = 15	37 (47) *n* = 15	37 (59.75) *n* = 16	37 (53) *n* = 13

^1^ Other = other cancer diagnoses than acute lymphoblastic leukemia (ALL) outside the central nervous system.
